# Loop-mediated isothermal amplification (LAMP) shield for Arduino DNA detection

**DOI:** 10.1186/s13104-018-3197-9

**Published:** 2018-02-01

**Authors:** Aldrik H. Velders, Cor Schoen, Vittorio Saggiomo

**Affiliations:** 1 Laboratory of BioNanoTechnology at Wageningen University and Research, Bornse Weiland 9, 6708 WG Wageningen, The Netherlands; 2 BioInteractions and Plant Health, Wageningen Plant Research, P.O. Box 69, 6700 AB Wageningen, The Netherlands

**Keywords:** Nucleic acid detection, Loop mediated isothermal amplification, Arduino instruments, Portable

## Abstract

**Objective:**

Loop-mediated isothermal amplification (LAMP) of DNA is gaining relevance as a method to detect nucleic acids, as it is easier, faster, and more powerful than conventional Polymerase Chain Reaction. However, LAMP is still mostly used in laboratory settings, because of the lack of a cheap and easy, one-button device that can perform LAMP experiments.

**Results:**

Here we show how to build and program an Arduino shield for a LAMP and detection of DNA. The here described Arduino Shield is cheap, easy to assemble, to program and use, it is battery operated and the detection of DNA is done by naked-eye so that it can be used in field.

**Electronic supplementary material:**

The online version of this article (10.1186/s13104-018-3197-9) contains supplementary material, which is available to authorized users.

## Introduction

Detection of genetic material such as DNA or RNA is one of the privileged analytical methods for confirming the presence of parasites, viruses, bacterial infections, or for example for food contaminations. When the amount of DNA or RNA is too low to be detected, their amplification is required. For long time the polymerase chain reaction (PCR) did the lion’s share as methodology to amplify DNA [1]. However, since its first description in 2000 by Notomi’s group [2], loop-mediated isothermal amplification (LAMP) has become one of the favorite methods for the detection of target DNA or RNA [3]. LAMP concerns an isothermal amplification, thus does not require the different temperature zones needed for the PCR. In addition, compared to PCR, LAMP is faster, sturdier, and less prone to inhibiting substances. Samples can be used without any prior purification like whole blood or extracts, its reagents can be freeze dried and are stable for months at room temperature [4]. Thanks to all these advantages, LAMP is the perfect candidate for in-field and point-of-care analysis. LAMP has been successfully used for example in the detection of malaria [5, 6], tuberculosis [7, 8], capripoxviruses [9], salmonella [7], many other infectious diseases [10] and beyond [11] in laboratories setting.

The effort is, nowadays, in fabricating cheap portable instruments for the use of LAMP in remote areas or third world countries, as commercial instruments are too expensive, not portable or difficult to be operated by untrained personnel. Recently, portable LAMP devices have been published, using heating blocks and cellphone cameras for real time detection [12, 13], paper based LAMP [14] and even battery-less LAMP systems [15]. However, the fabrication of such instruments is somehow still restricted to scientists with an electronic engineering background and not easily usable by untrained personnel.

Our goal is to fabricate a K.I.S.S (keep it simple, silly) instrument for LAMP analysis

The instrument should be:

- Cheap and easy to fabricate without extensive knowledge or expertise in electronic engineering;
- Portable and battery operated;
- Easily to modify or upgrade;
- One click instrument, usable by untrained personnel;
- Based on open electronics and open-source programs;
- The programming should be kept simple enough to be modified ad hoc.

## Main text

### Methods

The schematics for the electronics and the components needed for the Arduino LAMP as well as the software code can be found in the supplementary information. Schematics were drawn using Fritzing (http://fritzing.org) and licensed under CC Attribution-ShareALike. Master mix ISO_001 was obtained from Optigene (http://www.optigene.co.uk/). The concentration used was ISO_001 15 μL, Primermix 2 μL (Table 1), water 6 μL, gBlock 10–6 2 μL for a total of 25 μL.

**Table 1:** Primer concentrations used for the LAMP experiment

Primer	Work stock (µM)	Final conc. (µM)	1 ×
P_s_pA_new_F3	100	0.2	0.05
P_s_pA_B3	100	0.2	0.05
P_s_pA_FIP	100	0.8	0.2
P_s_pA_BIP	100	0.8	0.2
P_s_pA_LoopF	100	0.4	0.1
P_s_pA_LoopB	100	0.4	0.1
HyClone			1.3
Total			2

We used the gBlock BS2121 of *Pseudomonas syringae peponis* (Psp)

Pseudomonas_syringae_BS212:

AGGCAGTGCTGACGTACGCTCAGCTCAATGAGTCCGCCGACAAAATTGCCGACATATTGCTTCGCAAGGATGTGCAGCCCGGTGATGTCGTCGGTATCTGCATGATGCGCTCCGAGTGGCAGGTTGCAGCGCTCTTGGGCGTACTCAAAGCCGGTGCGTGCTACCTCTCGATCGACTGTGCGTCGCCTGCCGAGCGTCGTGACTGGCTGCTGGAAGAGGCGGACGTCAAGTGGGCACTGATCGATGAGAGTGCTCCGCCTCTACGTGATGCAACCTCAACGCTGTTGATCGGG

LAMP primers:

P_s_pA_new_F3 TGACGTACGCTCAGC

P_s_pA_B3 CACTCTCATCGATCAGTGC

P_s_pA_FIP CAGATACCGACGACATCACCGCGACAAAATTGCCGACATAT

P_s_pA_BIP ATGATGCGCTCCGAGTGGACAGTCGATCGAGAGGTAG

P_s_pA_LoopF CTGCACATCCTTGCGAAG

P_s_pA_LoopB GCTCTTGGGCGTACTCAA

The designed double-stranded synthetic genomic gene fragment (gBlock) contains the target sequence for Psp. All oligonucleotides and gBlock were synthesized by Integrated DNA Technologies (https://eu.idtdna.com/site). Oligonucleotides were re-suspended in Hyclone water (GE Healthcare Life Sciences, https://www.gelifesciences.com), gBlocks were re-suspended in 1 × TE Solutions pH 7.5 (Integrated DNA technologies).

## Results

**Fig. 1:**
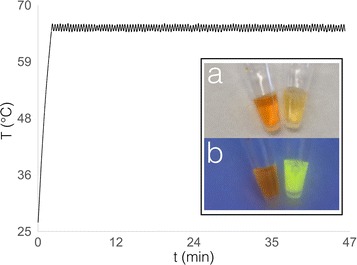
Heating profile of the PDMS heating block during the LAMP experiment. In the insert, end point SYBR green detection of the successful LAMP amplification (right vial) and the negative control (left vial), in **a** ambient light and **b** under a portable 365 nm UV lamp

We tested our Arduino LAMP shield for the amplification and detection of the Pseudomonas syringae gBlock. The LAMP enzyme mastermix was mixed with the six primers and synthetic target DNA (gBlock) in a final volume of 25 μL. When the green LED turned on, indicating that desired temperature has been reached, the Eppendorf tube was inserted in the heating block and the LAMP amplification was carried on until both LED’s on the Arduino shield turned on. The heating block reaches the desired temperature of 65.5 °C in 2 min and keeps a constant temperature of 65.5 ± 1.4 °C for the whole duration of the experiment (Fig. 1).

The LAMP enzyme mastermix used (Optigene) already contains the EvaGreen fluorescent dye, however its concentration is not enough for a simple detection by eye. When the sample was screened for a melting curve with a benchtop LAMP instrument (Genie III) it revealed the amplified product with a melting point at 90 °C, proving that the amplification worked correctly (Additional file 1). The LAMP reaction for the amplification of the Pseudomonas syringae gBlock is a standard DNA amplification used in our laboratory. The Arduino LAMP shield on this specific DNA amplification performed similarly as the benchtop system Genie III. We also tested an end point detection with SYBR green, and this latter gave an eye detectable signal (Fig. 1, insert). There are also other molecules that can be used either for end-point or real-time eye detection of the LAMP reaction [16]. In fact, it should be noted that being the PDMS transparent, the real-time LAMP can be performed by naked-eye as well.

The cost of the components for building an Arduino LAMP shield, thus without the Arduino board and the batteries, is as low as 8 € when using European retailers and less then 1.5 € when purchasing the parts from Chinese retailers.

It is also important to notice that the Arduino board is an extremely modular micro controller, and multiple add-ons can be added to the LAMP shield. For example a solar panel for recharging the batteries, or components for recording metadata during the LAMP experiments in the field, such as, external temperature, humidity, light, time, GPS position and record everything on an SD card.

In conclusion, we have developed a cheap, portable and battery operated, simply assemblable, easily usable, open-hardware, open-source Arduino LAMP shield for the detection of DNA. Temperature, experimental time can be easily adjusted by changing two variables in the open source code. The heating block, made out of PDMS, can be fabricated in-house and it is versatile as almost everything can be used as a mold. We envision this research spreading even more the use of LAMP for the detection of genetic materials in-field and in third world countries.

### Discussion

We focused on a simple, yet powerful, open electronics micro controller board: Arduino, which started as a do-it-yourself board for the maker community, it is becoming more and more used in laboratories around the world [17]. In this context, Arduino shields are commercially available plug-and-play add-ons for the Arduino micro controller and they work by simply plugging them on in the Arduino board. Commercially available Arduino shields add functionalities, for example, ethernet, WiFi, GPS and GSM, LCD, motors, capacitive touchpad and so on. Using the same idea of plug-and-play, in this communication, we describe the first Arduino LAMP shield.

**Fig. 2:**
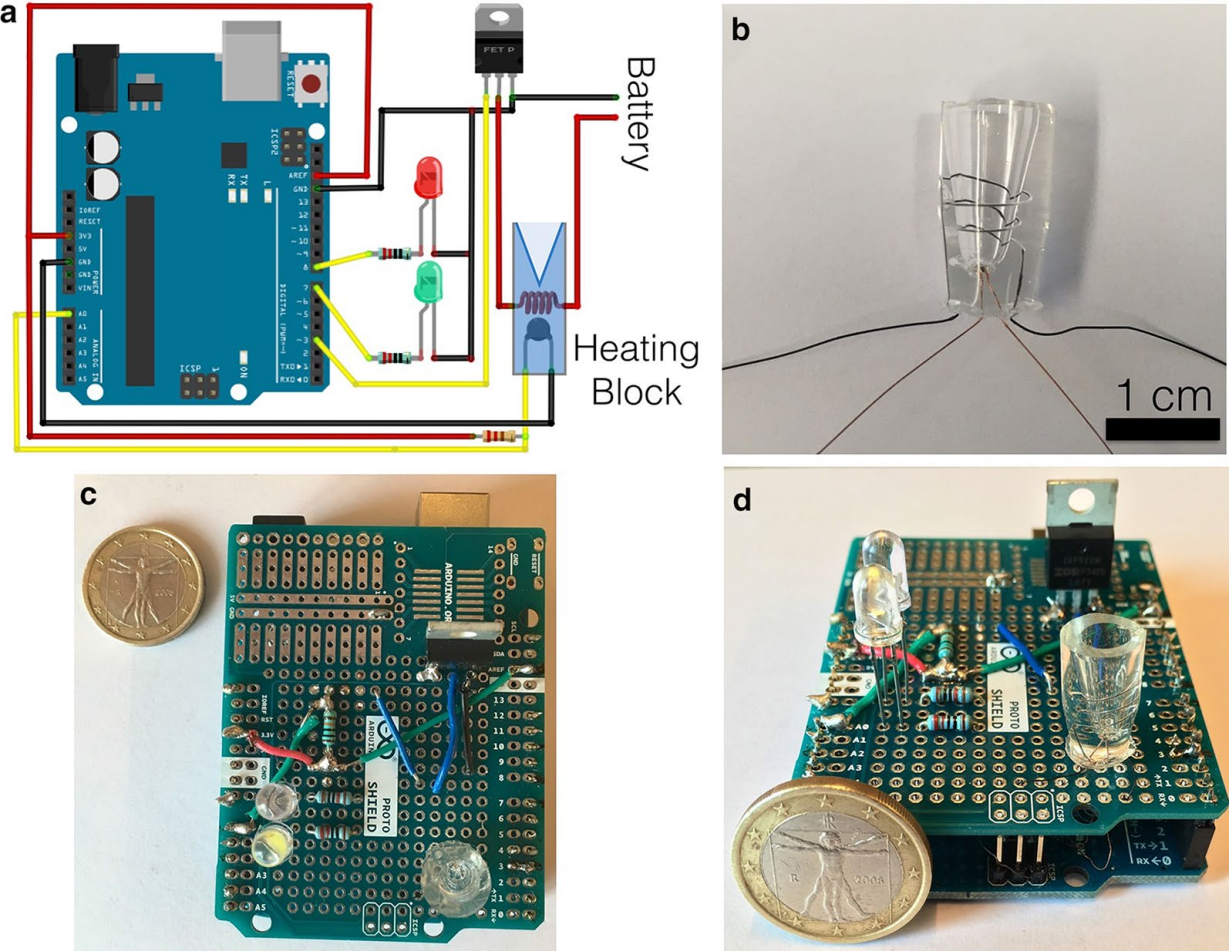
**a** Schematic for the connection of the electronics on the Arduino board. **b** PDMS heating coil, the nichrome resistance wire is a spiral near the PCR vial; the thermistor for controlling the temperature of the heating block is on the bottom of the vial. **c**, **d** Pictures of the final Arduino LAMP shield

We used a, so called prototype shield: a plug-and-play perforated board shield that fits on top of the Arduino for fabricating the Arduino LAMP shield. We keep the electronics to the basic, using only a MOSFET (metal–oxide–semiconductor field-effect transistor), for changing the voltage, thus the heating of the heating block by the use of an external battery, two LEDs for visual feedback, a few resistances and a heating block. A schematic of the LAMP shield is depicted in Fig. 2a; the detailed explanation, list of materials as well as the full schematics can be found in Additional file 1.

For the fabrication of the heating block, we opted for polydimethylsiloxane (PDMS) as material for the block, and a Nichrome wire for the heating part of it (Fig. 2b). Recently, we showed how a PDMS device can be heated using a resistance nichrome wire [18]. The PDMS heating block can be easily fabricated in the lab (or at home) using a PCR tube as template. As the PCR tube is used as template, the heating block fits perfectly the PCR tube, achieving high thermal transfer to the tube. A thermistor is inserted inside the PDMS heating block for controlling the temperature. PDMS is versatile and any Eppendorf or vials can be used as template for the mold, and in addition to this, the PDMS can be also used for fabricating microfluidic heating blocks. Although we used a PDMS heating block, the LAMP shield presented here can be used for any heating block, for example aluminum or peltier element based blocks. The LAMP shield here, is proposed as a single-shot experiment for using it as analytical yes-or-no tool in the field, so only one heating block has been attached to the board. However, multiple blocks can be used, naturally, to the detriment of the battery.

The Arduino code, in a few words, controls the thermistor and heats the coil until it reaches 65 °C, or any other temperature set by the user in the variable TARGET_TEMP, and it keeps the set temperature by switching on and off the heating coil. The green LED lights up when the temperature is reached, the red one is turned on when the feedback loop temperature is out of ranges, both lower or higher of the set temperature. After 45 min, both LEDs are turned on, signaling the end of the experiment. The whole program consist of less than 100 lines of code and it is attached and commented in the supplementary information of this communication.

The Arduino board can be powered in three different ways: via the USB port, the JACK socket or using directly the Vin pins. The JAPAN JACK socket and the USB ports are practicals as they can be used by external batteries.

The heating block is the most energy demanding part of the system and the Arduino maximal output of 40 mA is not enough to heat the nichrome wire. For this reason, it was decided to use a second external battery solely for the heating block. In terms of power consumption, during the LAMP experiment, the Arduino LAMP drains, on average, 35 mA at 5 V (0.175 W). Six 2500 mAh rechargeable AA NiMH provide 22.500 W to the Arduino board, which should allow the system to run continuously for more than 128 h.

During the heating process, the 5 cm of nichrome wire, drains 0.5 A, that, using a 7 V battery, accounts for 1.85 W of power. Therefore for the heating coil, we used a 3.7 V, 5100 mAh Li-Ion rechargeable battery that provides 18.8 W of power. The coil is switched between on and off for maintaining the heating block temperature at 65 °C, thus, the coil drains energy only half of the experiment time. This set-up used for the coil battery has a life of c.a. 20 h. LAMP amplification is also faster than the standard PCR, and it is not difficult to imagine that a successful fine-tuned LAMP amplification can be achieved in 20 min, doubling the lifetime of the battery.

## Limitations

Like all portable devices, the size and the capacity of the battery is the main limitation. However we can envision that in the future smaller and higher capacity batteries will be commercially available.

## Additional file

**Additional file 1.** Arduino LAMP shield electronics (Schematics and electronics used for the fabrication of the shield). Breakout of the prices (prices of single components). Fabrication of the heating block (Heating block fabrication using PDMS). Temperature test (temperature test of the heating block). Melting curve of the amplified gBlock (melting curve of the amplified product). Arduino LAMP shield source code (source code for the Arduino LAMP shield).

## Abbreviations

LAMP: loop mediated isothermal amplification
PCR: polymerase chain reaction
KISS: keep it simple silly
Psp: *Pseudomonas syringae peponis*
LED: light emitting diode
MOSFET: metal–oxide–semiconductor field-effect transistor
PDMS: polydimethylsiloxane
NiMH: nickel metal hydride
