# Supplementary material for: Loop-mediated isothermal amplification (LAMP) shield for Arduino DNA detection
Source: BMC Res Notes. 2018 Feb 1;11:93. doi: 10.1186/s13104-018-3197-9 (PMC5796575; doi:10.1186/s13104-018-3197-9)
Supplement: Supplementary file 1 — Additional file 1. Arduino LAMP shield electronics (Schematics and electronics used for the fabrication of the shield). Breakout of the prices (prices of single components). Fabrication of the heating block (Heating block fabrication using PDMS). Temperature test (temperature test of the heating block). Melting curve of the amplified gBlock (melting curve of the amplified product). Arduino LAMP shield source code (source code for the Arduino LAMP shield). [file 13104_2018_3197_MOESM1_ESM.pdf]

Supplementary information for

“Loop-Mediated Isothermal Amplification (LAMP) Arduino Shield”

Aldrik H. Velders, Cor Schoen, Vittorio Saggiomo

Arduino LAMP shield electronics

Breakout of the prices

Fabrication of the heating block

Temperature test

Melting curve of the amplified gBlock

Arduino LAMP shield source code

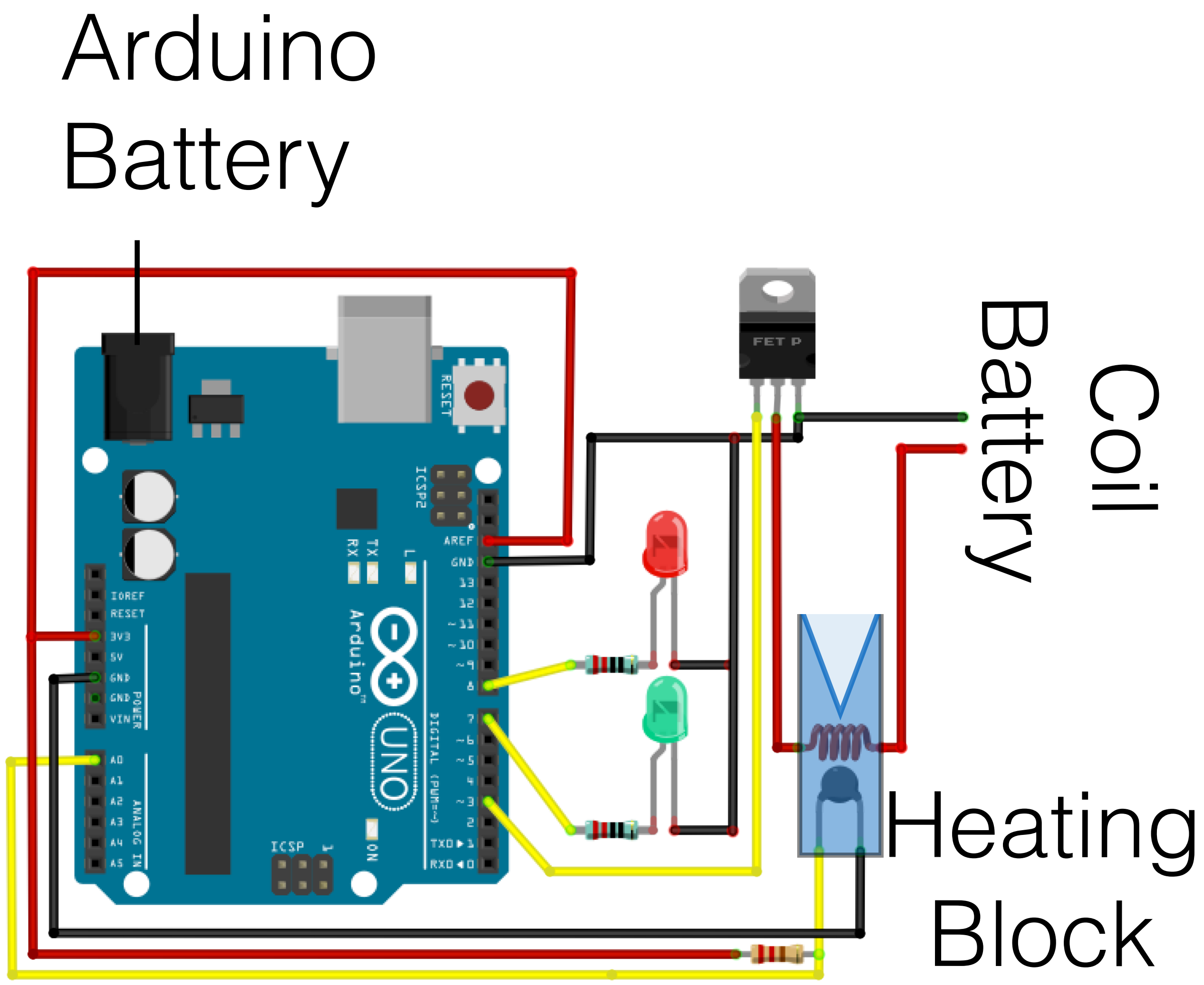

Figure 1. Schematic of the Arduino components for the LAMP Shield and their connection on the board.
MOSFET: International Rectifier IRF520NPBF N-channel MOSFET Transistor, 9.7 A, 100 V, 3-pin TO-220AB
Thermistor: EPCOS B57540G1104F Thermistor 100kΩ, 0.8 (Dia.) x 1.4mm
Resistors: 100kΩ for the heat calculation, and two 220Ω resistors for the LED
Heating coil: 5cm Nichrome 0.2mm diameter
Schematics drawn using Fritzing (http://fritzing.org) and licensed under CC Attribution-ShareALike

	RS-components	Aliexpress
Arduino UNO	21	3
prototype shield	4	1
EPCOS 100kΩ 18mW NTC Thermistor	3.3	0.3
3x resistors	0.1	0.01
2 LED	0.2	0.01
MOSFET	0.5	0.1
wires	0.2	0.01
heating block	0.2	
Li-ionBattery 3.7	30	7
6x AA NiMH batteries	27	4
case batteries	4	1

Figure 2. Breakout of the prices for the Arduino LAMP shield. Prices in Euro. RS-components is an European electronic components reseller, while Aliexpress is a Chinese one.

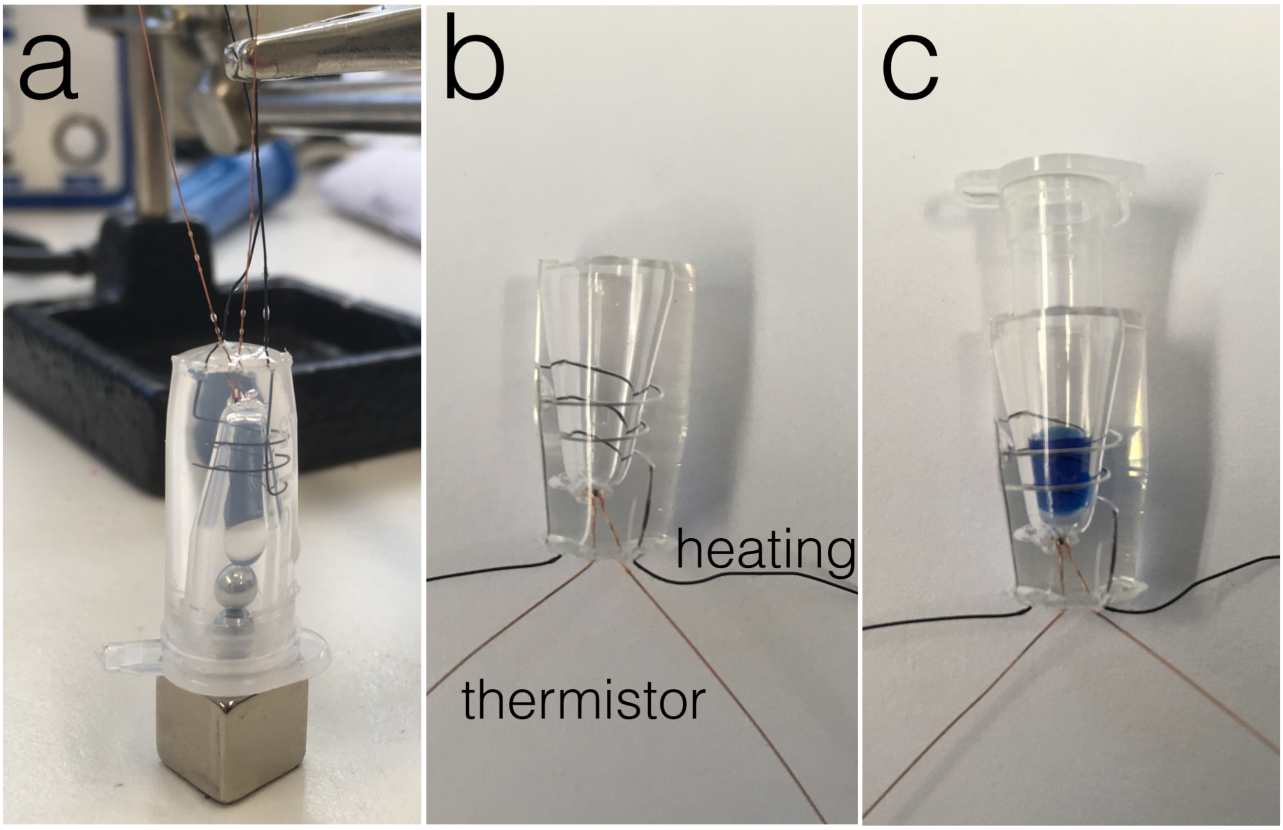

Figure 3. Fabrication of the heating block. a) the heating coil and the thermistor are suspended in liquid PDMS (10:1 PDMS and curing agent). An Eppendorf is used as mold. The PDMS is cured in an oven at 70 °C for two hours. b) The Eppendorf is removed and the heating block is ready to use. c) 25 uL of a blue dye are used to check the height of the heating coil.

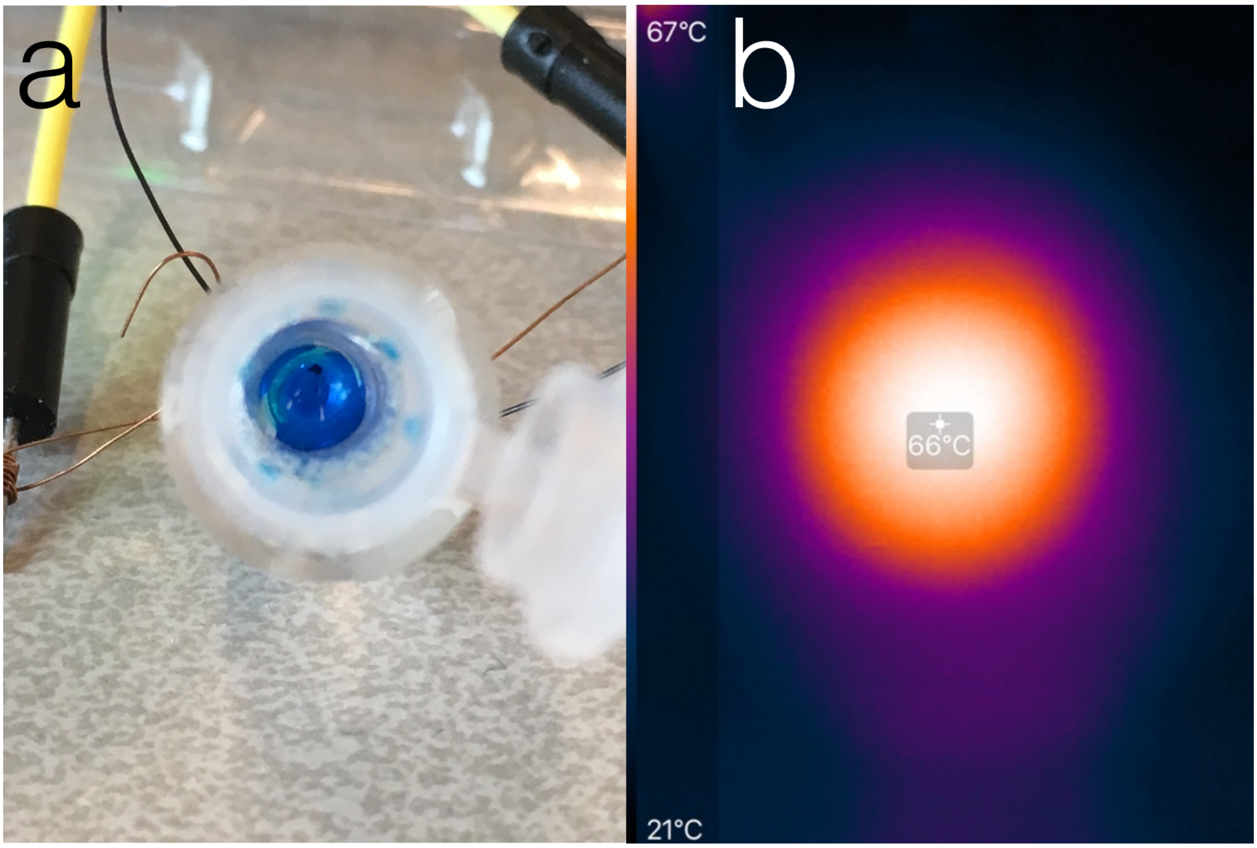

Figure 4. a) Heating block with an Eppendorf containing a blue dye. b) heat map of an open Eppendorf during the LAMP experiment.

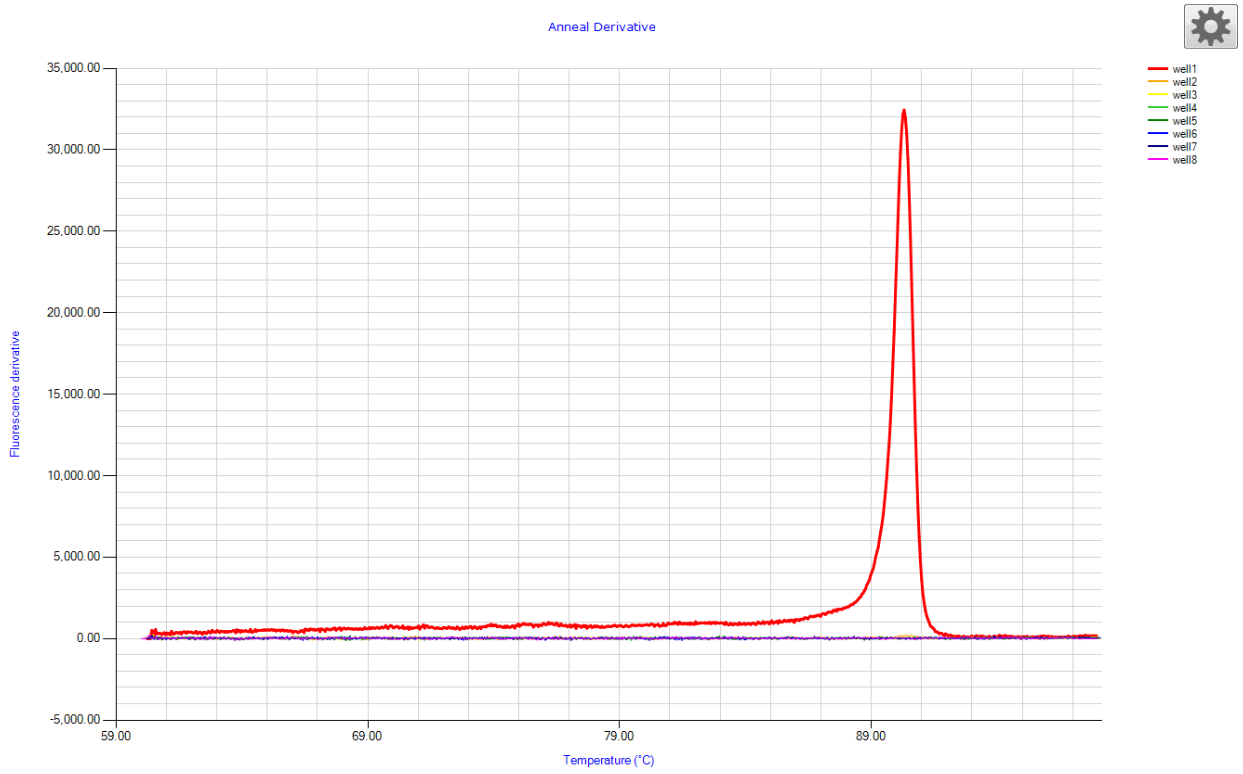

Figure 5. Melting curve experiment in the Genie III of the LAMP amplified gBlock product using the Arduino LAMP shield.

Arduino LAMP shield source code:

```
/*
  the thermistor code and math therein is based on https://learn.adafruit.com/thermistor/using-a-thermistor
  https://github.com/adafruit/Thermistor-test and the Steinhart–Hart equation for converting the resistance to temperature.

 Vittorio Saggiomo
  */

 /*
 // values that can be changed. Change the numbers for changing the operational temperature and time of the LAMP
 #define TARGET_TEMP 65.5
 #define MINTEMP 63
 #define MAXTEMP 68
 // time of the experiment in minutes
 #define TIMEmin 45
  */

 const float TARGET_TEMP = 65.6;
 const float MINTEMP = 63;
 const float MAXTEMP = 68;
 // time of the experiment in minutes
 #define TIMEmin 45


 // thermistor block
 // which analog pin to connect
 #define THERMISTORPIN A0
 // resistance at 25 degrees C
 #define THERMISTORNOMINAL 100000
 // temp. for nominal resistance (almost always 25 C)
 #define TEMPERATURENOMINAL 25
 // how many samples to take and average, more takes longer
 // but is more 'smooth'
 #define NUMSAMPLES 4
 // The beta coefficient of the thermistor (usually 3000-4000)
 #define BCOEFFICIENT 3950
 // the value of the 'other' resistor
 #define SERIESRESISTOR 100000


 // 0 Voltage setting in cycle zone C and if stable zone temp is exceeded.
 unsigned const int Temp0 = 0;
 // de- or increment of voltage setting if temp is off (zone B,D&F)
 unsigned const int HeatAdjust = 10;
 // tolerated difference between current and desired temp
 const float HeatTolerance = 0.1;
 // Initial value of the PWM signal sent to the thermistor gate
 int Set = 0;

 // the pin for controlling the MOSFET - heating coil
 #define heatingPin 5
```

```
// the pin of the green LED
#define greenLED 7

// the pin of the red LED
#define redLED 8

//converts the time from minutes to milliseconds
float TIME = TIMEmin*60000;

// this setup uses 30/40mA

int samples[NUMSAMPLES];

void setup(void) {
  // for the arduino serial monitor
  Serial.begin(9600);
   // pinmodes
   pinMode(heatingPin, OUTPUT);
   pinMode(greenLED, OUTPUT);
   pinMode(redLED, OUTPUT);
}


void loop(void) {
  uint8_t i;
  float average;
  // take N samples in a row, with a slight delay
  for (i=0; i<NUMSAMPLES; i++) {
   samples[i] = analogRead(THERMISTORPIN);
   delay(10);
  }
  // average all the samples out
  average = 0;
  for (i=0; i<NUMSAMPLES; i++) {
     average+=samples[i];
  }
  average /= NUMSAMPLES;
  // convert the value to resistance
  average = 1023 / average - 1;
  average = SERIESRESISTOR / average;

  // "steinhart" is the temperature reading converted in °C
  float steinhart;
  steinhart = average / THERMISTORNOMINAL;     // (R/Ro)
  steinhart = log(steinhart);                  // ln(R/Ro)
  steinhart /= BCOEFFICIENT;                   // 1/B * ln(R/Ro)
  steinhart += 1.0 / (TEMPERATURENOMINAL + 273.15); // + (1/To)
  steinhart = 1.0 / steinhart;                 // Invert
  steinhart -= 273.15;                         // convert to C

  // delay between the measuerements, in milliseconds
  delay(1000);

  // print the seconds and the temperature in the serial monitor
  Serial.print(millis()/1000);
  Serial.print(",");
```

```
  Serial.print(steinhart);
  Serial.print(",");
/*
    // if the temperature is lower than variable TEMP the program switches on the heating coil, if higher than variable TEMP then it switches it off.
    // In the serial monitor it prints 0 if the coil is off, and 1 if the coil is on
    if (steinhart >= TARGET_TEMP) {
   Serial.print("0,");
   analogWrite(heatingPin, 0);
 } else {
    Serial.print("1,");
    // heatingpin 100 = 370mA, 130=500mA, 150=580mA for a 5cm 200um nichrome coil
    analogWrite(heatingPin, 130);
 }

*/

// void checkHeating(float TARGET_TEMP, int Set){
float CurrentT = steinhart;
if (CurrentT <= (TARGET_TEMP - HeatTolerance)){
Set += HeatAdjust;
if (Set > 255){
 Set = 255;
}
} else {
if (CurrentT >= (TARGET_TEMP - HeatTolerance)){
  Set = 0;
} else {}; };

analogWrite(heatingPin, Set);
Serial.println(Set);


 // LED user feedback. If the temperature is between MINTEMP and MAXTEMP, the program turns on the green LED, in the other cases, it turns on the red LED
 if (steinhart > MINTEMP && steinhart < MAXTEMP) {
   digitalWrite(greenLED, HIGH);
   digitalWrite(redLED, LOW);
 }
 else  {
   digitalWrite(greenLED, LOW);
   digitalWrite(redLED, HIGH);
 }

// LED user feedback. After TIME both LEDs are turned on.
if (millis() > TIME ){
  digitalWrite(greenLED, HIGH);
  digitalWrite(redLED, HIGH);
}
else  {
}


}
```
